# Influence of geographical location and outdoor meteorological parameters on indoor humidity environment in rural residential buildings during the Plum Rains Season in the hot summer and cold winter region

**DOI:** 10.1371/journal.pone.0293181

**Published:** 2023-10-23

**Authors:** Yecong He, Jifei Zhou, Huaican Liu, Xiaofeng Zhang, Huan Zhou, Ke Wen, Jie Sun, Qi Deng

**Affiliations:** 1 School of Energy and Power Engineering, Changsha University of Science and Technology, Changsha, P. R. China; 2 State Key Laboratory of Air-Conditioning Equipment and System Energy Conservation, GREE Electric Appliances Inc. of Zhuhai, Zhuhai, P. R. China; Southwest Jiaotong University, CHINA

## Abstract

Plum Rains Season (PRS) has the typical characteristics of outdoor air temperature dramatic changes and high air humidity in the hot summer and cold winter region in China. When the outdoor temperature rises rapidly during PRS, the building envelope surface temperature is probably lower than the indoor air dew point temperature, resulting in moisture condensation. This paper evaluates the influence of geographical location and outdoor meteorological parameters on the indoor humidity environment. The effects of critical parameters such as altitude, average temperature, relative humidity, total precipitation, total precipitation days, atmospheric pressure, and wind speed on the building envelope moisture condensation in nine typical cities in the hot summer and cold winter region were simulated and analyzed. The results show that the Condensation Frequency (*CF*_*n*_) in the western, central, and eastern regions reached the highest in April, May, and June, respectively. Among the nine typical cities, Changsha has the highest Condensation Risk (*CR*). In addition, the altitude, total precipitation, and atmospheric pressure have little effect on the indoor humidity environment, and it is not directly related to *CR*; The average temperature and total precipitation days were not related to *CR* in the western and eastern regions and positively correlated with *CR* in the central region; The wind speed was positively correlated with *CR* in the western and central regions and negatively correlated in the eastern region; The relative humidity can affect the indoor humidity environment and moisture condensation on the inner surface of walls, when the relative humidity increases, the *CR* increases.

## 1 Introduction

China’s hot summer and cold winter region mainly refer to the middle and lower reaches of the Yangtze River and its surrounding areas. The climate is characterized by high temperature and high humidity in summer, cold and wet in winter, minor daily temperature differences, ample annual precipitation, and less sunshine. At the end of spring and the beginning of summer every year, there will be a unique climate phenomenon in this region—PRS (the regular wet and rainy season from late March to early June), the climate is hot, rainy, humid, outdoor temperature and often abrupt changes. When the outdoor air temperature rises sharply, the temperature of the inner surface of the wall rises slowly due to the heat storage effect of the building envelope, and the inner surface temperature may be lower than the dew point temperature of the indoor air, and condensation occurs at this time [1].

Condensation on building walls could lead to a deterioration of the thermal insulation performance of the building envelope [1], resulting in air condensation [2], wall material damage [3], wall mold [4], the indoor air quality deteriorates, and is harmful to the human body [5]. Moreover, the risk of moisture condensation on the inner surface of walls in the hot summer and cold winter region is much higher than in any other climate region in China [1]. To prolong the service life of the building and provide a suitable living environment for the occupants. Many scholars have studied the indoor thermal and humidity environment of the buildings.

Natural ventilation is an effective way to improve indoor air quality and the thermal environment. Zhu et al. [6] analyzed and evaluated the indoor thermal environment through the operating temperature and believed that the thermal performance of existing rural residential buildings was poor and the indoor thermal environment was not ideal. Tang et al. [7] believed that ventilation is used to maintain acceptable indoor air quality and thermal comfort and put forward a control strategy to change the window opening factor. Gu et al. [8] believed that occupants can change the indoor thermal environment by opening windows. Yin et al. [9] proposed a method for measurement and evaluation of indoor air quality in naturally ventilated residential buildings. Cao et al. [10] proposed energy-saving renovation evaluation methods suitable for natural ventilation buildings. Pilar et al. [11] summarised the variation in the surface condensation risk due to the reduction of the ventilation rates.

The building envelope significantly influences the indoor thermal and humidity environment. Jiayu et al. [12] combined with the "Envi-met" model and the "TRNSYS" model to predict the impact of the window-to-wall ratio on indoor cooling energy demand in southern Hunan. Qin [13] established a coupled model associated with heat and moisture transfer in the building envelope based on multi-regional moisture and heat flow to evaluate the impact of humidity on indoor air quality and building energy consumption. Yu et al. [14] believed that the building envelope is a multi-layer porous structure that transfers heat and moisture to balance the indoor and outdoor temperature difference and water vapor partial pressure difference. Liu et al. [15] thought that opening windows is by far the simplest means of ventilation. It is also crucial for energy efficiency and for creating a comfortable indoor environment. Joowook et al. [16] proposed a simplified heat and moisture transfer model for building envelopes. Ivanov et al. [17] revealed a numerical study of the risk of condensation over the exterior wall of a typical residential room, and the resulting surface and dew point temperature distribution are derived over the simulated exterior wall. Zhigang et al. [18] studied the dynamic heat transfer characteristics of the wall with heat pipes implanted in transition season and its influence on the indoor thermal environment. Xie et al. [19] studied the performance of capillary ceiling cooling panels on ceiling surface temperature and indoor thermal environment.

Building envelope materials can effectively prevent the risk of condensation. Xie et al. [20] used a new composite hygroscopic material for building envelopes to evaluate its moisture buffering performance and studied its effect on indoor thermal and humidity environment. Ziye et al. [21] found that building envelopes integrating heat reflective coatings (HRC_s_) and phase change materials (PCM_s_) can block solar radiation from the outdoors and help reduce indoor temperatures. NR et al. [22] found that rice husk-incorporated foam concrete wall panels as a thermal insulation material in buildings can have a major influence on the thermal conductivity of the wall panels. Fantucci et al. [23] found a novel aerogel-based insulating coating, particularly suitable for the mitigation of thermal bridges and the prevention of condensation risk. Bendouma et al. [24] found that using biobased insulation may delay and even prevent the risk of interstitial condensation. Xue et al. [25] found inappropriate thermal insulation measures may exacerbate the humidity within the building envelope and further induce condensation and mould growth, which threatens the building structural safety and indoor air quality. Pruteanu et al. [26] analysed the occurring of condensation risk and its location in the outer wall made of wood-concrete blocks by the Glaser method, for different situations of arrangement and composition of the layers.

Previous studies mainly focus on the indoor thermal environment. However, there are few studies on indoor humidity environment. The main objectives of this study are as follows:

1. To determine the geographical location and outdoor meteorological parameters in nine typical in the hot summer and cold winter region in China during PRS, which is lacking in existing research;
2. To investigate the relationship between condensation and geographical parameters(altitude) and outdoor meteorological parameters (temperature, relative humidity, total precipitation and total precipitation days, atmospheric pressure, and wind speed) and determine which factors have a significant impact on indoor humidity environment; and
3. Two indicators, condensation frequency and condensation risk, are used for evaluation.

## 2 Methods

**Fig 1:**
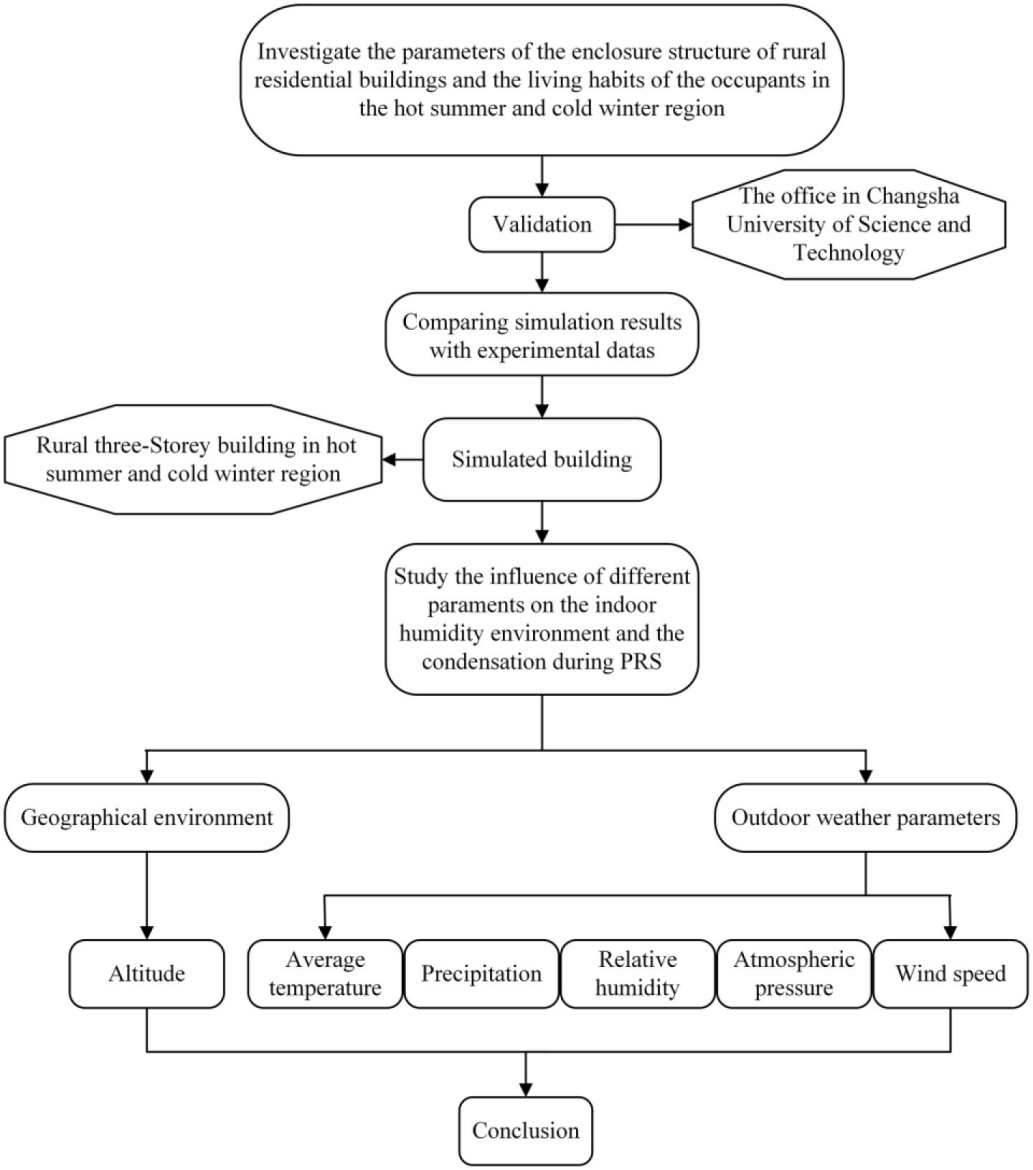
Technical route.

In this paper, the effectiveness of the research method is verified by simulation and measurement of the indoor thermal and humidly environment in the office at the Changsha University of Science and Technology. In order to study the influence of geographical environment and outdoor meteorological parameters on indoor humidity and condensation during PRS. Using EnergyPlus as a simulation tool, a typical rural three-storey building was built in a hot summer and cold winter region. The technical route is shown in Fig 1.

### 2.1 Main parameters

In the Chinese building regulations [27], condensation on the room surface would form when the interior surface temperature is lower than the air dew point temperature. Two indicators, condensation frequency *CF*_*n*_ and condensation risk *CR*, are used for evaluation. The difference between *CF*_*n*_ and *CR* is that *CF*_*n*_ is used to evaluate the moisture condensation on the inner surface of the wall, and *CR* is used to evaluate the condensation risk of the entire room.

#### 2.1.1 Condensation frequency

The condensation frequency *CF*_*n*_ [28] is an indicator that expresses the ratio of the total hours when condensation has occurred to the 2184 h (April-June) for the wall surface *n* in the building and has 6 values(eastern wall, southern wall, western wall, northern wall, ceiling, floor) for each time step. When studying the condensation on the inner surface of the wall, the *CF*_*n*_ can be used to intuitively see the condensation on the six inner surfaces of the room, which is conducive to judging which inner surface of the wall is more serious.

Taking the area of a wall surface *n* (= 1, 2, …, 6 surfaces) as *A*_*n*_ and the occurrence (judgment = 1) or non-occurrence (judgment = 0) of condensation on the wall surface *n* during PRS time step t (= 1, 2, …, 2148 hours) as *IC*_*n*,*t*_. The condensation frequency *CF*_*n*_ for wall surface *n* was defined using Eq (1).


$$CFn=\frac{\sum_{t=0}^{2184}ICn,t}{2184}\times 100\;(n=1,2,\ldots ,6)$$ (1)

#### 2.1.2 Condensation risk

The condensation risk *CR* [28] is an indicator calculated from the area average *CF*_*n*_ of all wall surfaces in a building. The total area of the 6 surfaces of the rural building simulation model is 144m^2^. When studying the indoor humidity environment, the *CR* can be used to see the overall condensation situation of the room intuitively. The condensation risk *CR* during PRS is defined using Eq (2).


$$CR=\frac{\sum_{n=1}^{6}CFnAn}{\sum_{n=1}^{6}An}$$ (2)

0≤*CR*≤1. When *CR* > 0, it indicates that condensation occurs inside the building. That is, the indoor wet environment of the building deteriorates. The larger the value, the more serious the condensation phenomenon of the inner surface of the building wall.

## 3 Validation

**Fig 2:**
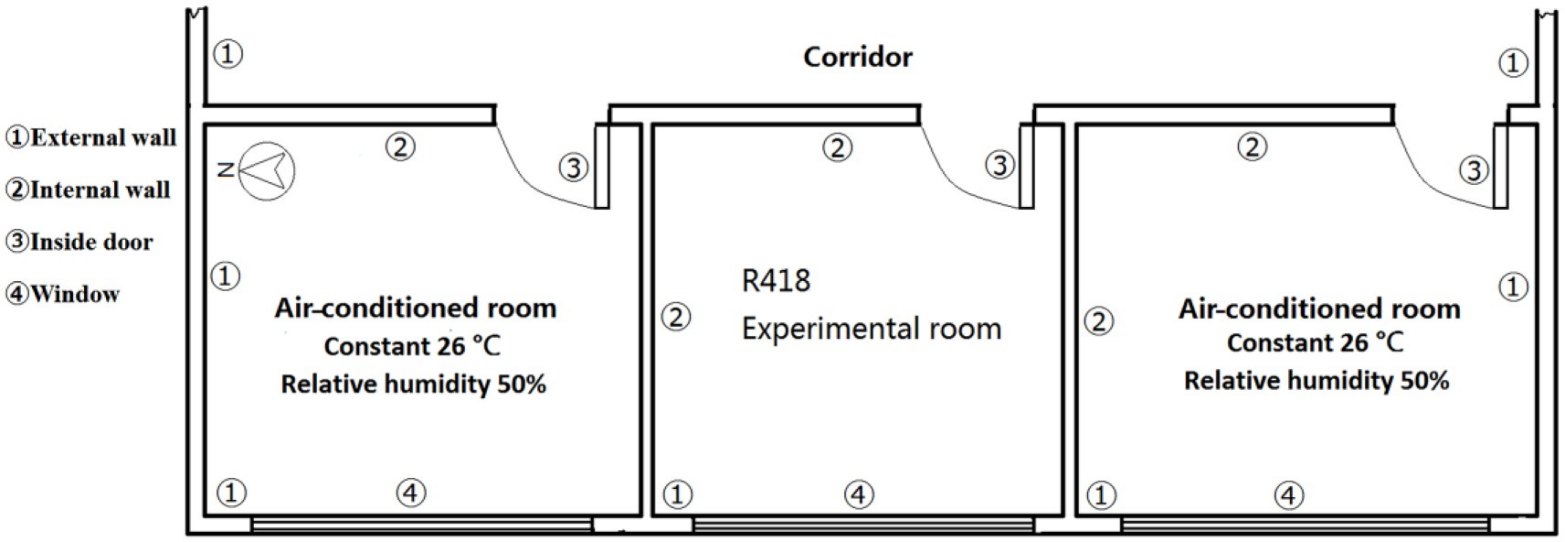
The building envelope.

The effectiveness of the research simulation method is verified by the field measurement of the thermal and humid environment of a university office on R418 of Changsha University of Science and Technology (Fig 2). The area of the room is 6m×8m, and the floor height is 4m. The adjacent room is set as an air-conditioned room in the simulation. The specific parameter settings are given in Table 1. The building envelope parameters are given in Table 2 according to the actual building conditions.

**Table 1:** Parameter setting [29].

Door	Single-layer solid wood door with a thickness of 25.3mm
**Sunlight transmittance**	0.67
**Shading factor of the dark cloth curtain**	0.65
**window frame correction factor**	1.07
**Location correction coefficient in Changsha**	1.4
**Window**	6mm single-layer glass window with a heat transfer coefficient of 5.7

**Table 2:** Envelope parameter of the building.

Building Structure	Material name and thickness	Thermal-conduction resistance (m^2^•K)/W	Heat transfer coefficient, W/(m^2^•K)	Index of thermal inertia
**External wall**	Heavy mortar clay (240mm) + The pure gypsum board (10mm) +Polystyrene foam material (60mm) +The pure gypsum board (10mm)	1.616	0.564	3.579
**Internal wall**	Cement mortar (20mm) + Ceramsite concrete (180mm) + Cement mortar (20mm)	0.43	1.515	2.69
**Roof**	Cement mortar (20mm) + Reinforced concrete (200mm) + Polystyrene (46mm) +Cement mortar (20mm)	1.522	0.595	2.837
**Ground**	Cement mortar (20mm) + Gravel or pebbles (40mm)	0.047	N/A	0.589
**Floor**	Cement mortar (20mm) + Reinforced concrete (100mm) + Cement mortar (20mm)	0.108	2.963	1.406

### 3.1 Measurement

#### (1) Measurement parameters

Indoor and outdoor temperature, indoor and outdoor humidity ratio, and internal surface temperatures of eastern, southern, western, and northern internal walls, ceilings, and floors in the R418 room.

#### (2) Measurement instruments

Experimental instruments for measurement are shown in Table 3.

**Table 3:** Experimental device.

Measurement parameters	Measuring instrument	Measurement accuracy
Outdoor temperature and humidity ratio	Automatic temperature and humidity recorder (TH22R-EX)	0.1°C, 1.5% RH
Inner wall surface temperature	non-contacting infrared thermometer (AS842A-0-1)	0.1°C
Indoor temperature and humidity ratio	Mercury thermometer, Automatic temperature and humidity recorder (TH22R-EX)	0.1°C, 0.1°C,1.5% RH

#### (3) Measurement period

March 1st to March 30th, 2021 (to verify the validity of the simulation), continuous monitoring with one-hour measurement interval

### 3.2 Measurement sites

**Fig 3:**
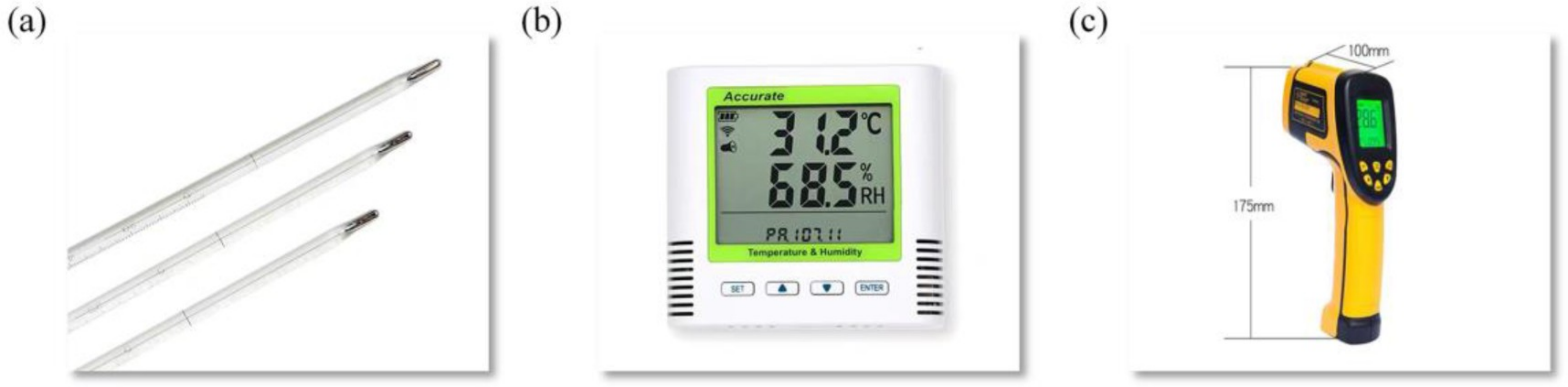
Mercury thermometers, automatic temperature and humidity recorder, non-contact infrared thermometer.

The measuring instruments (Fig 3) are arranged as follows:

1. In the R418 room, hang three mercury thermometers at 1.5*m* from the ground, 3*m* from the west wall, and 2*m*, 4*m*, and 6*m* from the north wall, respectively, to measure the indoor temperature every hour.
2. An automatic temperature and humidity recorder are placed in the middle of the floor to measure the hourly indoor temperature and hourly indoor humidity, and another is placed outside to measure the hourly outdoor temperature and hourly outdoor humidity.
3. Use a non-contact infrared thermometer to measure the hourly temperature of each measurement point in Fig 3. The arrangement and measurement of other inner wall surfaces are the same as above.

**Fig 4:**
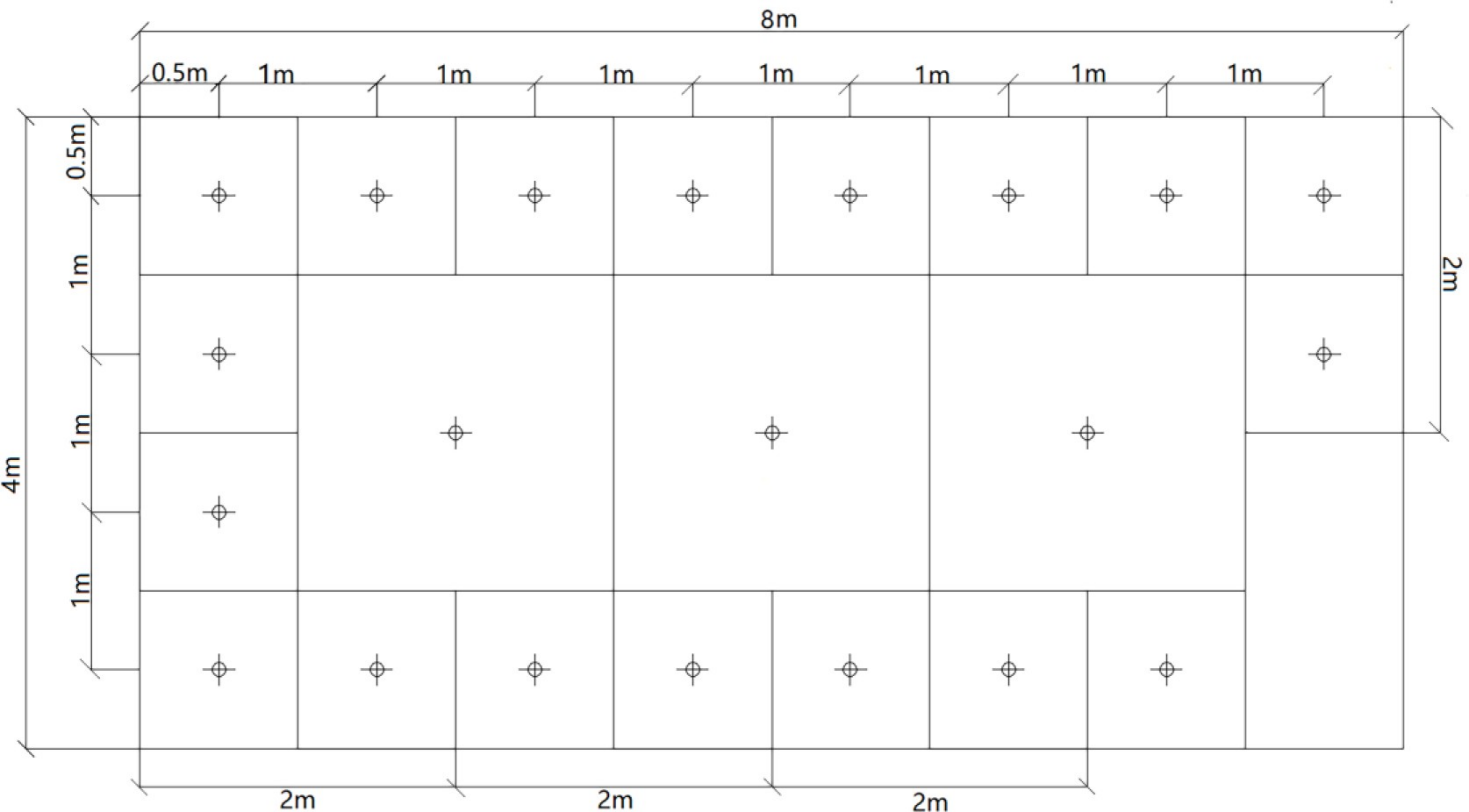
The layout of measurement locations on the eastern wall.

Considering the influence of the thermal bridge on the arrangement of measuring points, the measuring position and quantity of the inner surface of the building are determined. The number of sampling locations was reduced by dividing the rectangular section of the room into eighteen 1 *m*^2^ squares around the perimeter and three 2 x 2 *m* = 4 *m*^2^ squares in the center, the lower right corner is the door on the east wall, as shown in Fig 4.

Fig 4 shows the layout of the measurement locations on the eastern interior wall. The eastern interior wall area is 32*m*^2^. Measurements were made at 21 measurement positions in the center of each square marked on the eastern inner wall.

**Fig 5:**
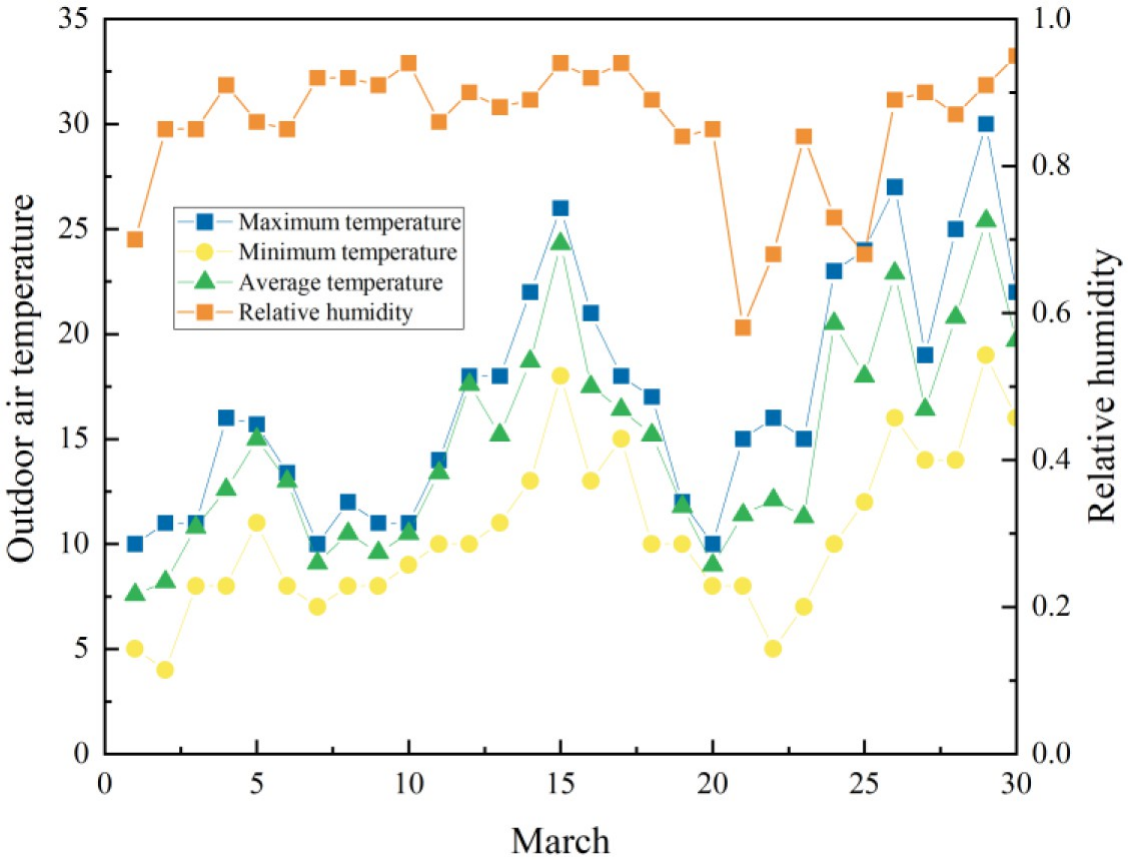
Measured outdoor temperature and relative humidity.

**Fig 6:**
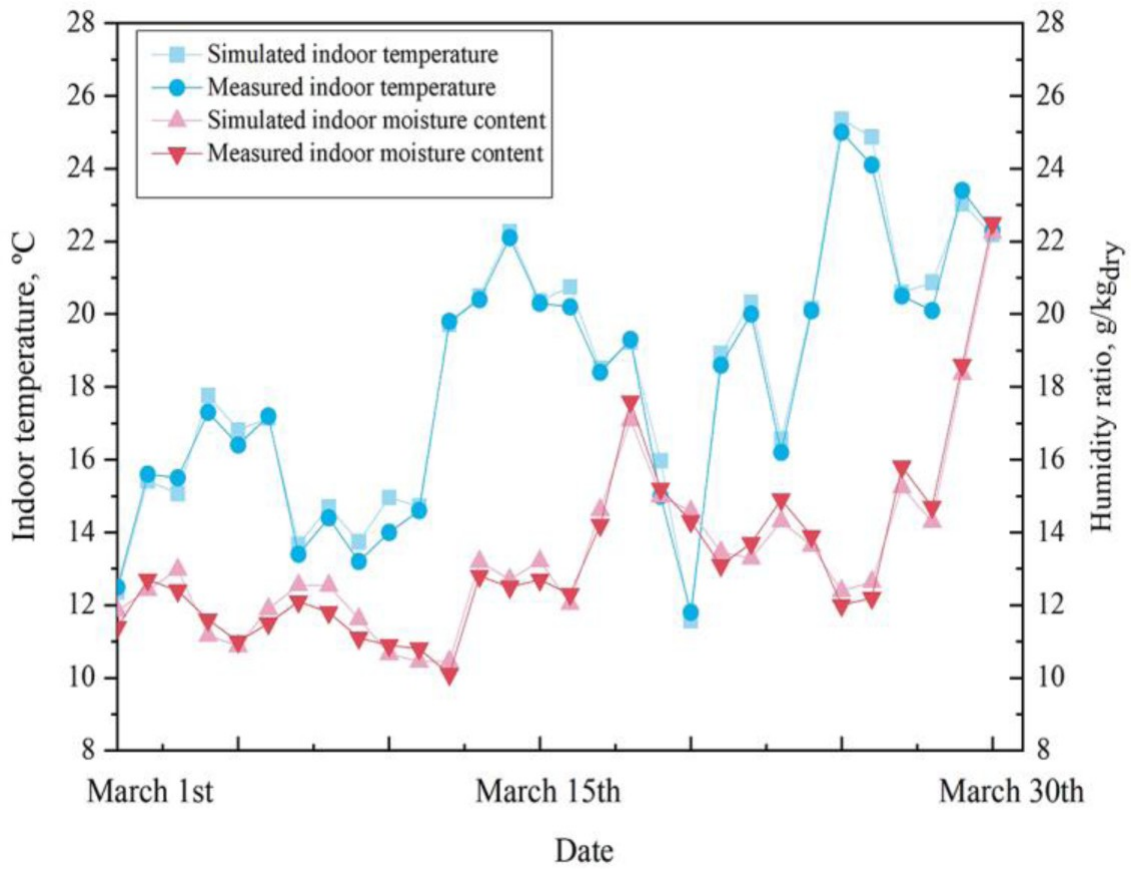
R418 from March 1st to March 30th, indoor temperature and humidity ratio in the room.

**Fig 7:**
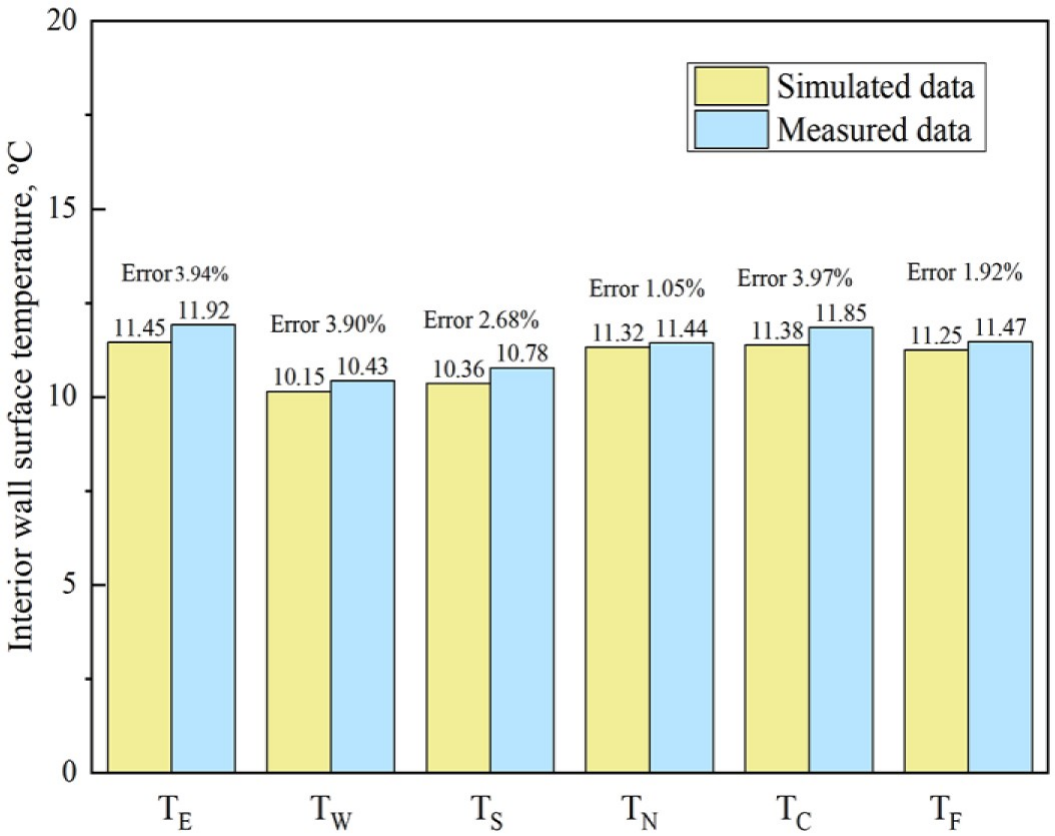
Interior wall surface temperature in R418 from March 1st to March 30th.

The outdoor meteorological parameters measured in March (Fig 5) were input into EnergyPlus for simulation, and indoor parameter simulation data was obtained. Simulated parameters were compared to the measured indoor parameters to determine analytical errors. The comparison of simulation results and experimental data are plotted in Figs 6 and 7.

Fig 6 shows that the indoor temperature measurement error is between 0.30% and 6.86%, and the indoor humidity ratio measurement error is between 1.10% and 6.25%. Fig 7 shows that the interior wall surface temperature measurement error is between 1.05% and 3.97%. The following reasons may cause the error: 1) The number of measurement positions is limited; 2) Improper measurement operation. All in all, the deviation of the measured value from the actual value is minimal.

## 4 Simulated building

### 4.1 Typical rural residential building

**Fig 8:**
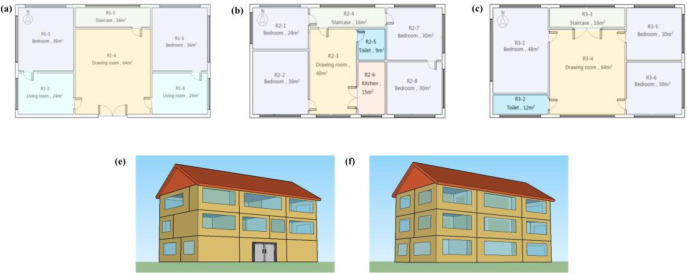
Rural three-storey building.

Rural three-storey building was built, as shown in Fig 8. The total construction area is 600*m*^2^, and the height is 3*m*, including nine bedrooms, two living rooms, three staircases, three drawing rooms, two toilets, one kitchen, and a total of twenty rooms. The model takes the top, middle and bottom floors, covering all conventional room types. Therefore, this model can be used as a representative building for predicting the condensation of rural buildings in the hot summer and cold winter region.

In this paper, the rural three-storey building and its parameters of the enclosure structure are applied to the nine typical cities. The building envelope parameters were obtained from the standard, which meets the Design Standard for Energy Efficiency of Residential Buildings in Hot Summer and Cold Winter Zone [30]. Taking Changsha as an example, the simulated meteorological parameters use the typical meteorological year data of Changsha. The room thermal and moisture load are shown in Table 4. The parameters of the enclosure structure are shown in Table 5. The door is a single-layer solid wooden door with a thickness of 25.3mm, and the window is a 6mm single-layer glass window with a heat transfer coefficient of 5.7 [29] and location correction coefficient of 1.4 in Changsha (Chengdu, Chongqing, Zunyi, Changsha, Wuhan, Nanchang, Shanghai, Nanjing, Hangzhou-1.5, 4.1, 3.8, 1.4, 3.1, 3.4, 3.3, 0.6, 3.5). The frame correction coefficient is 1.07, the solar transmittance is 0.67, and the shading coefficient is 0.6 for light-colored cloth curtains. The surface temperature of the inner wall and indoor dew point temperature will be obtained through numerical simulation.

**Table 4:** Room thermal and moisture load [29].

Types	Per capita humidity production, g/h	Per capita thermal production, W	Equipment power, W
**Bed room**	61	53	15
**Living room**	61	53	15
**Drawing room**	61	53	80
**Staircase**	61	53	10
**Toilet**	102	60	5
**Kitchen**	102	60	5

**Table 5:** Envelope parameter of the building.

Building Structure	Material name and thickness	Thermal-conduction resistance (m^2^•K)/W	Heat transfer coefficient, W/(m^2^•K)	Index of thermal inertia
**External wall**	Lime mortar (20mm) + Concrete perforated brick (220mm) + Bituminous vermiculite slate (40mm) +Lime mortar (20mm)	0.727	1.13	3.362
**Internal wall**	Cement mortar (20mm) + Ceramsite concrete (180mm) + Cement mortar (20mm)	0.43	1.515	2.69
**Roof**	Asphalt felt (15mm) + Lime mortar (20mm) +Bituminous vermiculite slate (40mm) +Reinforced concrete (120mm) + Lime mortar (20mm)	0.592	1.334	2.658
**Ground**	Asphalt concrete(40mm) + Gravel or pebbles (40mm)	0.064	N/A	0.997
**Floor**	Cement mortar (20mm) + Cellular concrete(200mm) + Reinforced concrete (130mm) + Cement mortar (15mm)	1.074	0.812	4.281

## 4.2 Typical cities

The hot summer and cold winter region are divided into western, central, and eastern regions. Nine cities are selected as typical cities to research. Chengdu, Chongqing, and Zunyi are selected in the western regions; Changsha, Wuhan, and Nanchang are selected in the central regions; Shanghai, Nanjing, and Hangzhou are selected in the eastern regions. The following are the geographical locations of typical cities (Table 6):

**Table 6:** The geographical environment of typical cities.

Regions	Cities	Latitude and longitude
**Western**	Chengdu	102°54′E~104°53′E 30°05′N~31°26′N
	Chongqing	105°11′E~110°11′E 28°10′N~32°13′N
	Zunyi	106°17′22″E~107°26′25″E 27°13′15″N~28°04′09″N
**Central**	Changsha	111°53′E~114°15′E 27°51′N~28°41′N
	Wuhan	113°41′E~115°05′E 29°58′N~31°22′N
	Nanchang	115°27′E~116°35′E 28°10′N~ 29°11′N
**Eastern**	Shanghai	120°52′E~122°12′E 30°40′N~31°53′N
	Nanjing	118°22′E~119°14′E 31°14′N~32°37′N
	Hangzhou	118°21′E~120°30′E 29°11′N~30°33′N

## 5 Simulation results and discussion

**Fig 9:**
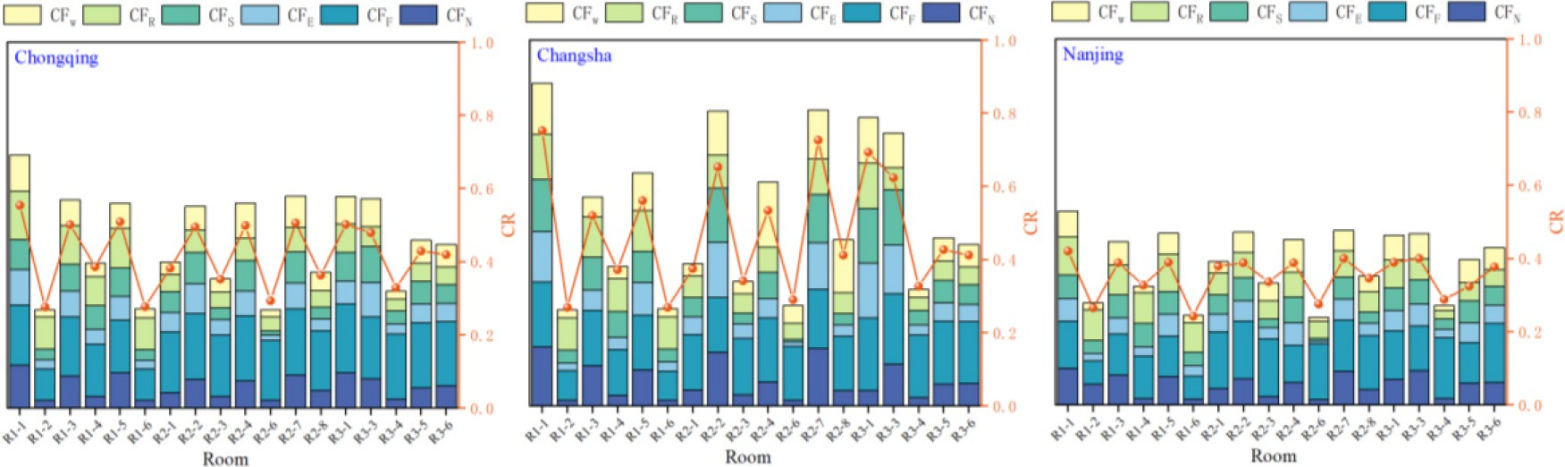
The *CF*_*n*_ and *CR* (except toilet R2-5 and R3-2) of all rooms in Chongqing, Changsha and Nanjing.

Based on the verified research methods, the indoor thermal and humidity environment and condensation conditions of rural buildings during the PRS period in nine typical cities were simulated. Among the nine cities, R1-1 has the most severe condensation. Due to manuscript space constraints, we listed Chongqing, Changsha, and Nanjing among the nine typical cities in the western, central, and eastern hot summer and cold winter region. The *CF*_*n*_ and *CR* of all rooms (except toilets R2-5 and R3-2) were studied. It can be seen from the Fig 9 that R1-1 has higher *CF*_*n*_ and *CR* than other rooms, so we chose R1-1 as our research object. We were taking the condensation frequency *CF*_*n*_ of Changsha in April, May, and June as example.

**Fig 10:**
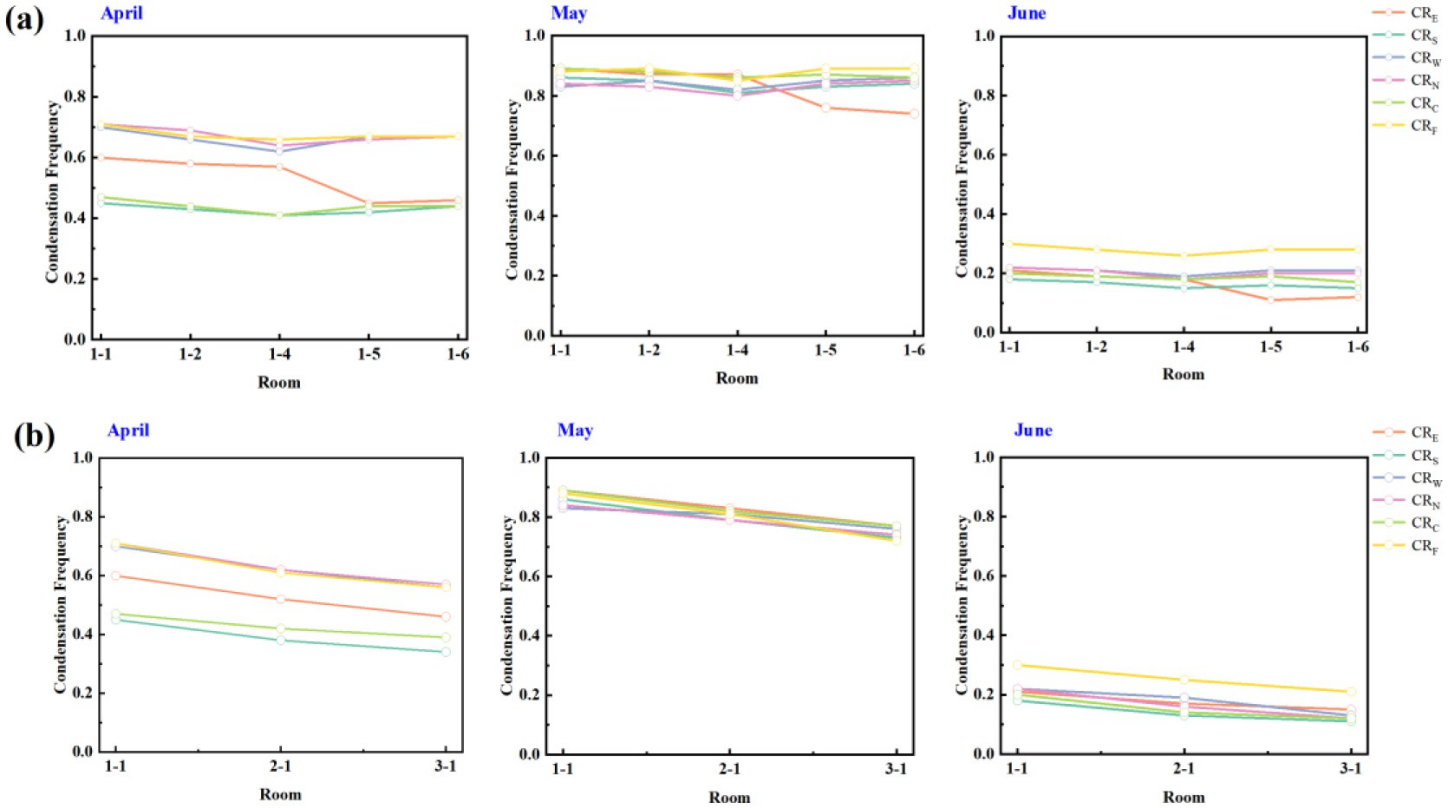
Horizontal and longitudinal comparison.

Fig 10(a) selected the *CF*_*n*_ of all rooms on the first floor (R1-1 R1-2 R1-4 R1-5 R1-6). The *CF*_*n*_ on the inner walls of the R1-1 is much more serious than that of the inner walls of other rooms. Fig 10(b) selected the *CF*_*n*_ of rooms (R1-1, R2-1, R3-1) located on the same horizontal line on different floors. As the number of floors increases, the *CF*_*n*_ on the inner surface of walls gradually decreases. According to the above, the R1-1 is selected as the research object of nine typical cities in the hot summer and cold winter region. Our detailed boundary conditions of the simulations and setting parameters are as follows (Tables 7–10).

**Table 7:** Initial building parameters.

Window-wall ratio	Exterior wall reflectivity	Window opening mode (on/off)	The main moisture source inside the room
0.5 [31]	0.45 [31]	Fully-opened	People (Occupant)

**Table 8:** Setting parameters of people.

Room	Schedule	number of people	Software instructions
R1-1	00:00~8:00	2	1
	08:00~12:00	0	0
	12:00~14:00	2	1
	14:00~24:00	0	0

**Table 9:** Setting parameters of lights.

Room	Schedule	Lighting power density (W/m^2^)	Software instructions
R1-1	0:00~8:00	6 [31]	0
	8:00~22:00		0.5
	22:00~24:00		1

**Table 10:** Setting parameters equipment.

Room	Schedule	Electrical equipment power	Software instructions
R1-1	0:00~8:00	520W [31]	0
	8:00~22:00		0.5
	22:00~24:00		1

**Fig 11:**
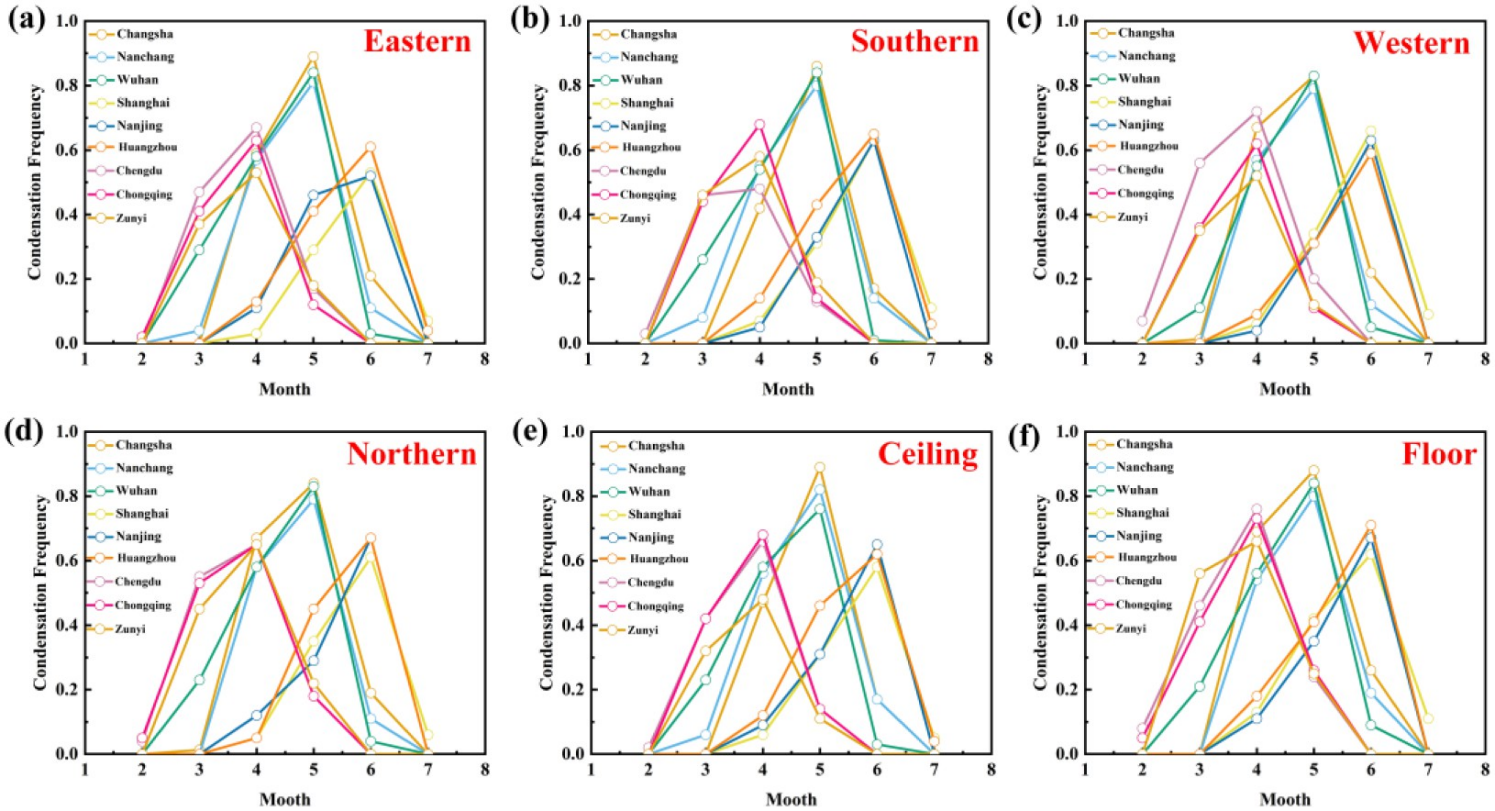
*CF*_*n*_ on the inner surface of room walls in nine typical cities in the hot summer and cold winter region, R1-1.

Fig 11 shows the variation of condensation frequency *CF*_*n*_ on the inner surface of room walls in nine typical cities in the hot summer and cold winter region, R1-1. 1) In the western region (Chengdu, Chongqing, Zunyi), the condensation on the inner surface of the wall was concentrated from February to May. The most serious condensation occurred in April, which *CF*_*n*_ ranged from 0.45 to 0.76. 2) In the central region (Changsha, Nanchang, Wuhan), the condensation on the inner surface of the wall was concentrated from March to June. The most serious condensation occurred in May, where *CF*_*n*_ ranged from 0.80 to 0.89. The average *CF*_*n*_ in May is above 0.8, which means that the condensation problem for the whole month exceeds 80%. 3) In the eastern region (Shanghai, Nanjing, Hangzhou), the condensation on the wall’s inner surface was concentrated from April to July. The worst condensation occurred in June, which *CF*_*n*_ ranged from 0.52 to 0.71. In contrast, the condensation on the inner surface of the wall in the central region is the most serious, and the condensation in the western and eastern regions is slightly relieved.

When the *CF*_*n*_ of all cities reached the maximum, The most serious condensation on the eastern inner wall is Changsha, with a *CF*_*n*_ of 0.89, and the lighter one is Nanjing, with a *CF*_*n*_ of 0.52. The most condensation on the southern inner wall is Changsha, with a *CF*_*n*_ of 0.86, and Chengdu with a lighter *CF*_*n*_ of 0.48. The most serious condensation on the western inner wall are Changsha and Wuhan, with a *CF*_*n*_ of 0.83, and the lighter city is Zunyi, with a *CF*_*n*_ of 0.52. The most serious condensation on the northern inner wall is Changsha, with a *CF*_*n*_ of 0.84, and the lighter city is Shanghai, with a *CF*_*n*_ of 0.61. The most serious condensation on the inner ceiling wall is Changsha, with a *CF*_*n*_, and the lighter city is Zunyi, with a *CF*_*n*_ of 0.48. The most serious condensation on the floor’s inner wall is Changsha, with a *CF*_*n*_ of 0.88, and the lighter city is Shanghai, with a *CF*_*n*_ of 0.62.

From the above simulation results, the *CF*_*n*_ in the western, central, and eastern regions reached the highest in April, May, and June, respectively. Among all the cities, Changsha has the most serious condensation on the inner surface of the walls. The effects of geographic location and outdoor meteorological parameters on the indoor humidity environment and moisture condensation in hot summer and cold winter region will be studied below.

### 5.1 Altitude

In the geographical sense, Altitude refers to the vertical distance above or below sea level of a particular place or geographical object on the ground. The western region of China has a higher altitude and is dominated by mountain plateaus; low mountains and hills dominate the central region; the eastern region has a lower altitude and is dominated by plains and mountains. The terrain is higher in the west and lowers in the east, with a stepped distribution.

**Fig 12:**
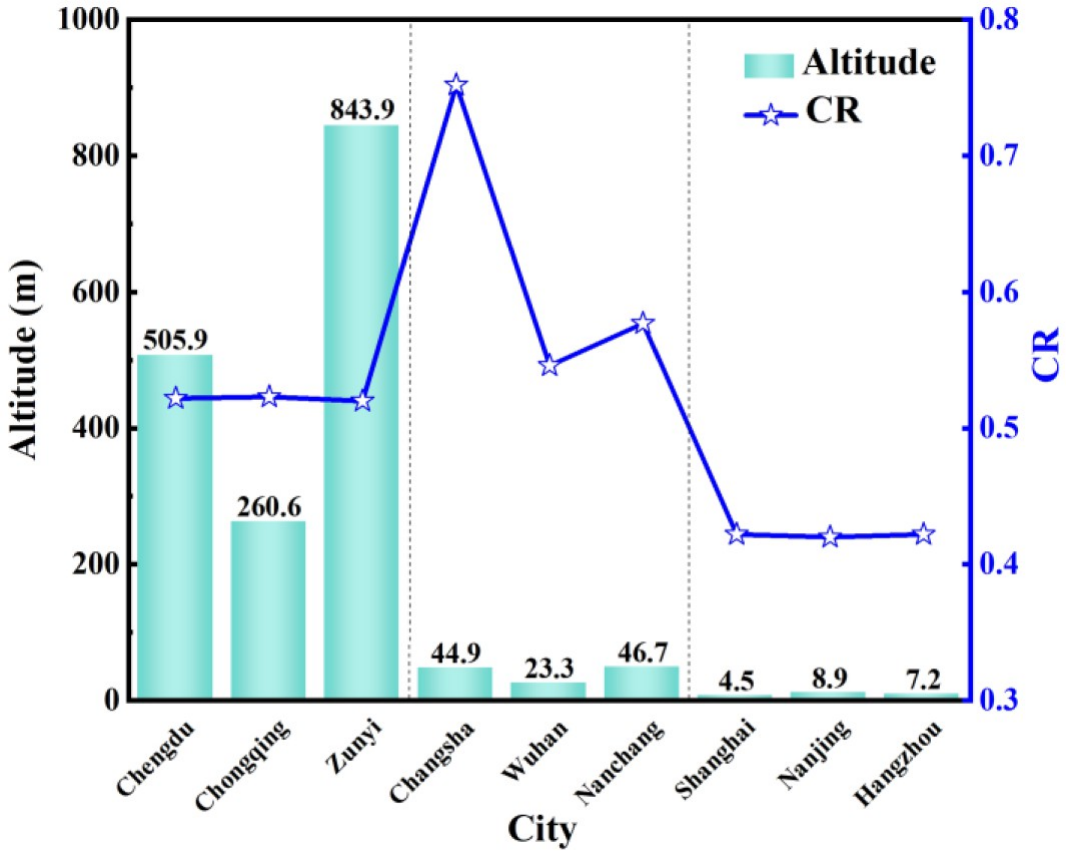
The altitude and *CR* of nine typical cities.

Fig 12 compares the altitude and condensation risk *CR* of nine typical cities in the hot summer and cold winter region. The altitude in the western region is between 260m and 850m, the central region is between 23m and 50m, and the altitude in the eastern region is less than 10m. The altitude in the western region is significantly higher than that in the central and eastern regions. The *CR* in the central region varies widely but is not directly related to altitude. In the western and eastern regions, the *CR* did not change much with increasing altitude. This is because water vapor pressure decreases with the increase of altitude more significantly than air pressure. The size of the vapor pressure of water reflects the content of water vapor in the wet air. At a specific temperature, the more significant the water vapor pressure, the more water vapor content in the air, and the more humid air. When the water vapor content in the wet air reaches the maximum limit, the vapor pressure of water at this time is called the saturated partial pressure of water vapor. When the ambient temperature continues to decline, condensation will occur. The temperature of the wet air only determines the partial pressure of saturated water vapor. In contrast, the partial pressure of unsaturated water vapor is related to the atmospheric pressure and determined by the actual atmospheric pressure. So altitude can determine whether the air is wet or dry. There was no direct relationship between altitude and *CR*. It is also affected by other geographical conditions.

### 5.2 Average temperature

**Fig 13:**
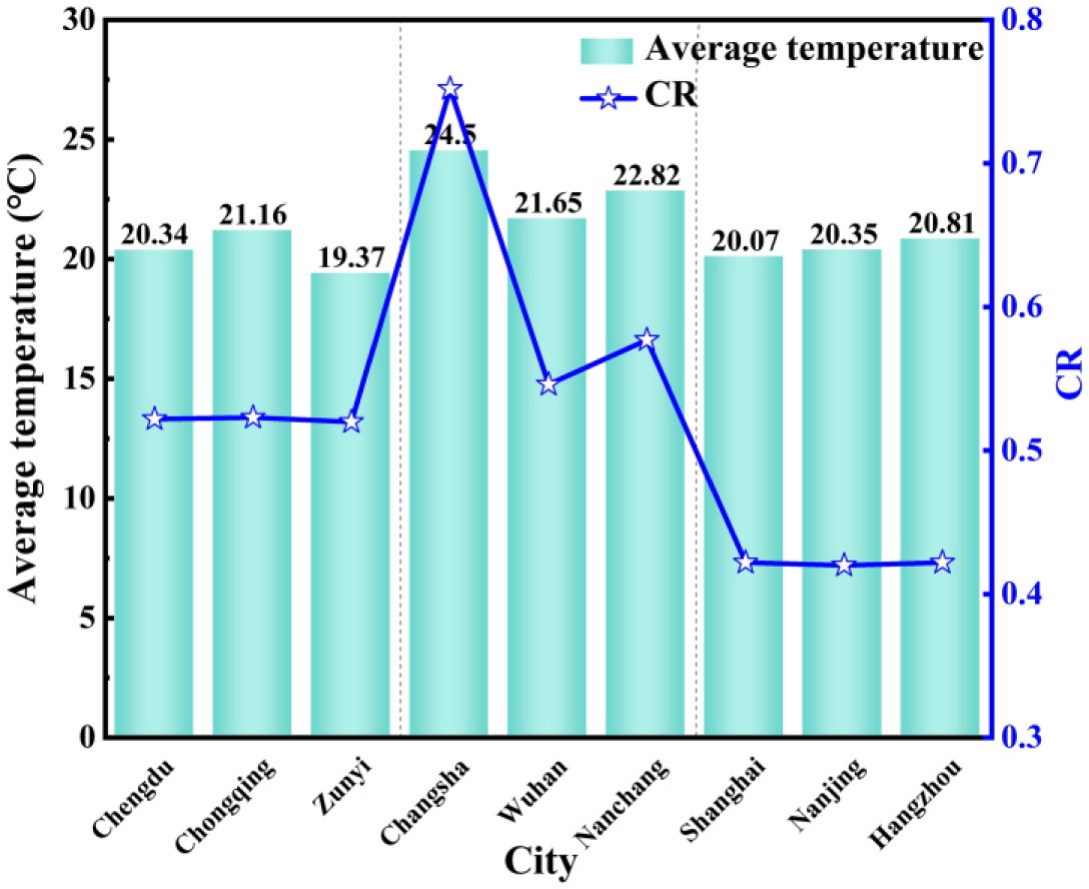
The outdoor temperature and *CR* from April to June.

Fig 13 shows the comparison chart of the average outdoor temperature and *CR* from April to June in nine typical cities. The average outdoor temperature in the central region is the highest, and Changsha has the highest average outdoor temperature of 24.5°C, followed by the western region. The average outdoor temperature in the eastern region has no significant change around 20°C. In addition, *CR* in the western region is higher than that in the eastern region. However the correlation between outdoor temperature and *CR* in the western and eastern regions is not significant, while the outdoor temperature in the central region is positively correlated with *CR*. It is mainly because the envelope structure with a thermal insulation layer increases wall’s thermal resistance, increases the thermal inertia index of the envelope structure, and reduces the overall heat transfer coefficient. When the outdoor temperature rises, the temperature of the inner surface of the wall rises slowly due to the heat storage effect of the thermal insulation wall. At this time, the time that the internal surface temperature of the wall is lower than the air dew point temperature is prolonged, and the *CR* increases. The condensation phenomenon is serious due to the increase of *CR*.

Due to the condensation on the inner surface of the floor is the most serious, the following will select the duration of condensation on the R1-1 floor on April 15th, May 15th, and June 15th for research.

**Fig 14:**
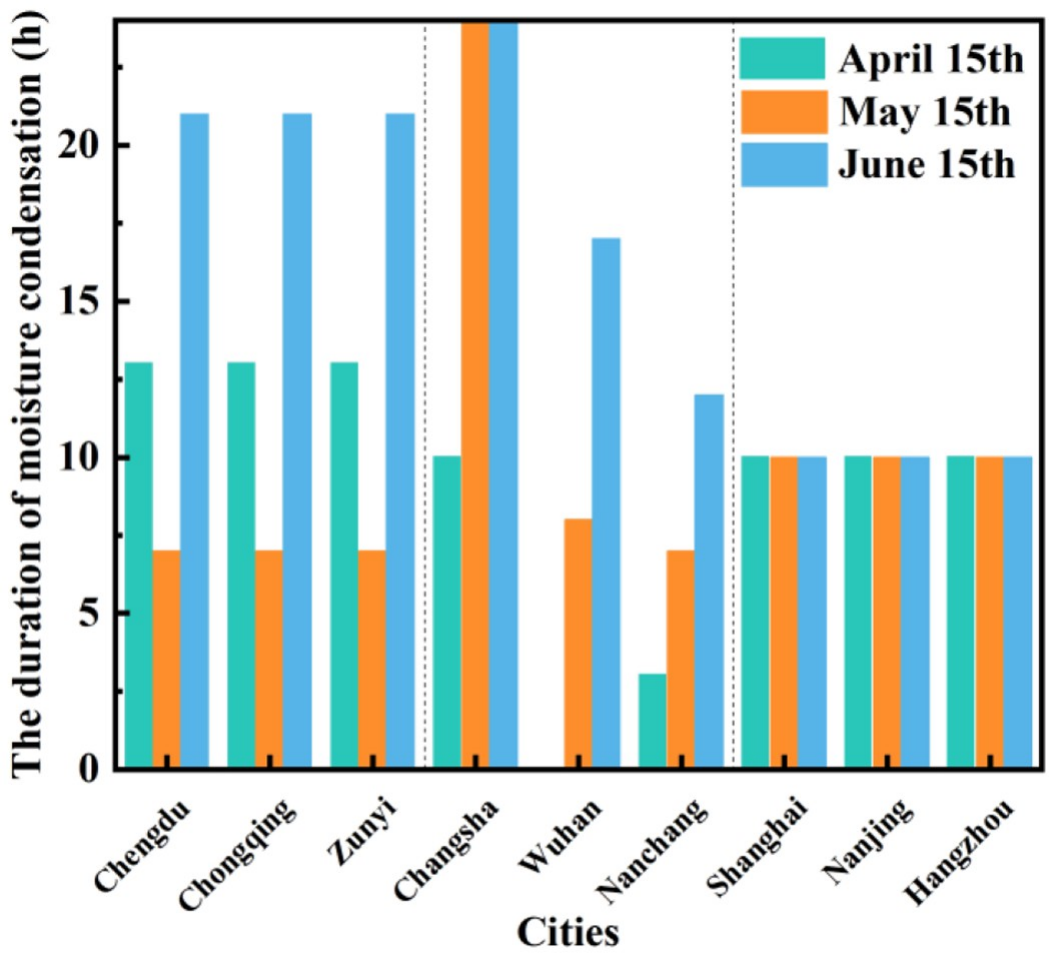
The duration of moisture condensation on April 14, May 15, and June 15.

When the indoor dew point temperature is higher than the temperature of the inner surface of the floor, condensation will occur at this time. After a period, when the indoor dew point temperature is lower than the temperature of the inner surface of the floor, the condensation will end. It can be seen from Fig 14 that the condensation duration in the western region was 13, 7, and 21 hours, respectively. The condensation duration in Changsha in the central region was 10, 24, and 24 hours, respectively; the condensation duration in Wuhan was 0, 8, and 17 hours; and the condensation duration in Nanchang were respectively 12, 3, and 7 hours, the condensation duration in the eastern region was all 10 hours. When the outdoor temperature increased (April 15 to June 15), the condensation duration in the western and eastern regions remained unchanged, and the condensation duration in the central region increased.

It shows that the average outdoor temperature in the western and eastern regions has no significant correlation with *CR*, and the outdoor temperature in the central region was positively correlated with *CR*. This is due to the higher outdoor temperature in the central region. When the air temperature rises, evaporation increases, the absolute humidity increases, and the water vapor reach saturation. When the temperature outside changes abruptly and drops, the air can no longer hold any more water vapor, and water vapor becomes over-saturated and condenses, so the *CR* is higher in central regions.

### 5.3 Relative humidity

**Fig 15:**
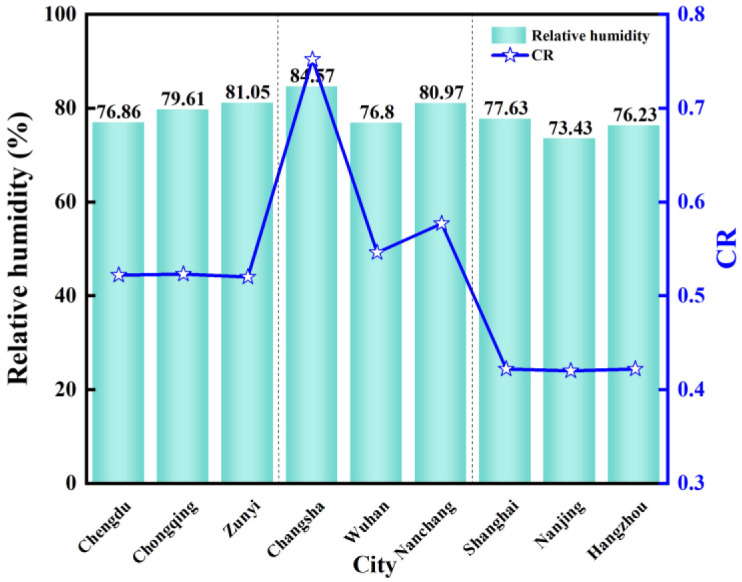
The relative humidity and *CR* from April to June.

Fig 15 shows the comparison chart of relative humidity and *CR* in nine typical cities from April to June. The relative humidity and *CR* in the central region were the highest, followed by the western region, and the lowest in the eastern region. Among them, Changsha has the highest relative humidity and *CR*. The average outdoor relative humidity is between 73% and 85%, and the *CR* reaches its maximum when the average outdoor relative humidity is 84.57%. Relative humidity refers to the percentage value of the actual water vapor density in the air and the saturated water vapor density at the same temperature. The dryness, humidity, and amount of water vapor in the air are close to saturation. It is not directly related to the amount of water vapor contained in the air. Usually, the temperature is inversely proportional to relative humidity. For example, dew in summer is due to the decrease in temperature at night, the ability of the air to dissolve water decreases, causing excess water to condense to form dew. In the northern winter, there is heating in the room, which increases the room temperature and reduces the relative humidity. At this time, a water basin will be placed in the room to increase the humidity. Therefore, when the temperature decreases, the time that the inner surface of the wall is lower than the air dew point temperature is prolonged, the *CR* and relative humidity increased.

**Fig 16:**
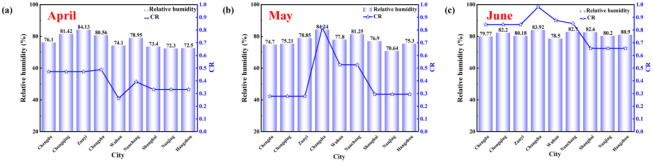
The relative humidity and *CR* in April, May, and June.

Fig 16 shows the average relative humidity and *CR* in April, May, and June. It can be seen from Fig 16(a) that the relative humidity in the western region in April was higher than that in the other two regions. The relative humidity in Chongqing and Zunyi is above 80%, except for Chengdu, where the relative humidity is 76.1%. In the central region, only Changsha is above 80%, while the relative humidity in the eastern region is around 72%, which is significantly lower than the other two regions. In addition, the *CR* in the western region is 0.472, which is higher than the other two regions. When the relative humidity increases, the *CR* increases. From Fig 16(b), the relative humidity in the western region in May decreased compared with that in April, while the relative humidity in the central region increased compared with that in April, and the relative humidity in Changsha reached 84.24%. In the eastern region, except for Nanjing, the relative humidity in Shanghai and Hangzhou increased to 76.9% and 75.35%. In addition, the *CR* in the western region decreased with the decrease of relative humidity to 0.279, while the *CR* in the central region increased greatly with the increase of relative humidity, and the *CR* in the eastern region decreased to 0.295. From Fig 16(c), the relative humidity in the western and central regions did not change much in June, but the relative humidity in the eastern region increased more than 80%. Overall, the *CR* in June increased significantly. At this time, anti-condensation measures need to be taken.

It shows that relative humidity affects the indoor humidity environment to a certain extent. Relative humidity was significantly positively correlated with *CR*. The higher the relative humidity, the higher the *CR*, and the condensation phenomenon is serious. This is because the condensation phenomenon is related to temperature and moisture content. The relative humidity is the ratio between the actual content of water vapor and the maximum possible content under certain temperature conditions. When the wet air with high relative humidity is in contact with the surface of the object below the dew point temperature, the air temperature decreases, and the water is separated, resulting in condensation.

### 5.4 Total precipitation and total precipitation days

**Fig 17:**
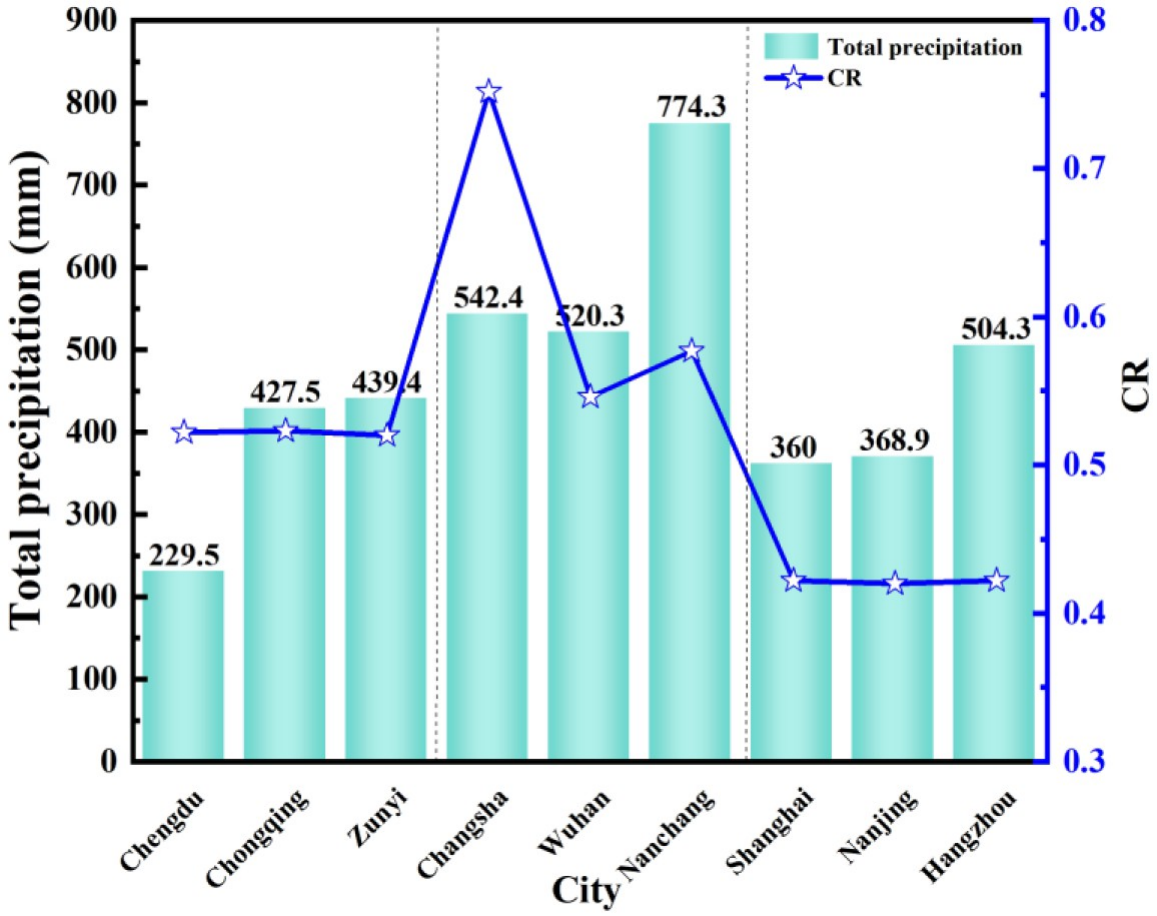
The total precipitation and *CR* from April to June.

Fig 17 shows the comparison chart of the total precipitation and *CR* of nine typical cities from April to June. The total precipitation in the central region is higher than in the other two regions. In the western region, the total precipitation except in Chengdu is as low as 229.5mm, and the total precipitation in Chongqing and Zunyi is about 400mm. In the central region, the total precipitation in Nanchang is 774.3mm, and the total precipitation in Changsha and Wuhan is about 520mm. In the eastern region, the total precipitation in Shanghai and Nanjing is only about 360mm. The total precipitation may be related to the geographical environment. When it rains, the outdoor water vapor condenses on the rainwater to reduce the humidity, and the indoor humidity is reduced. The total precipitation has an impact on the indoor humidity environment. In addition, when the total precipitation in Chengdu decreased and the total precipitation in Nanchang and Hangzhou increased, the *CR* did not change significantly. The total precipitation was not directly related to *CR*, which may be related to the total number of precipitation days.

**Fig 18:**
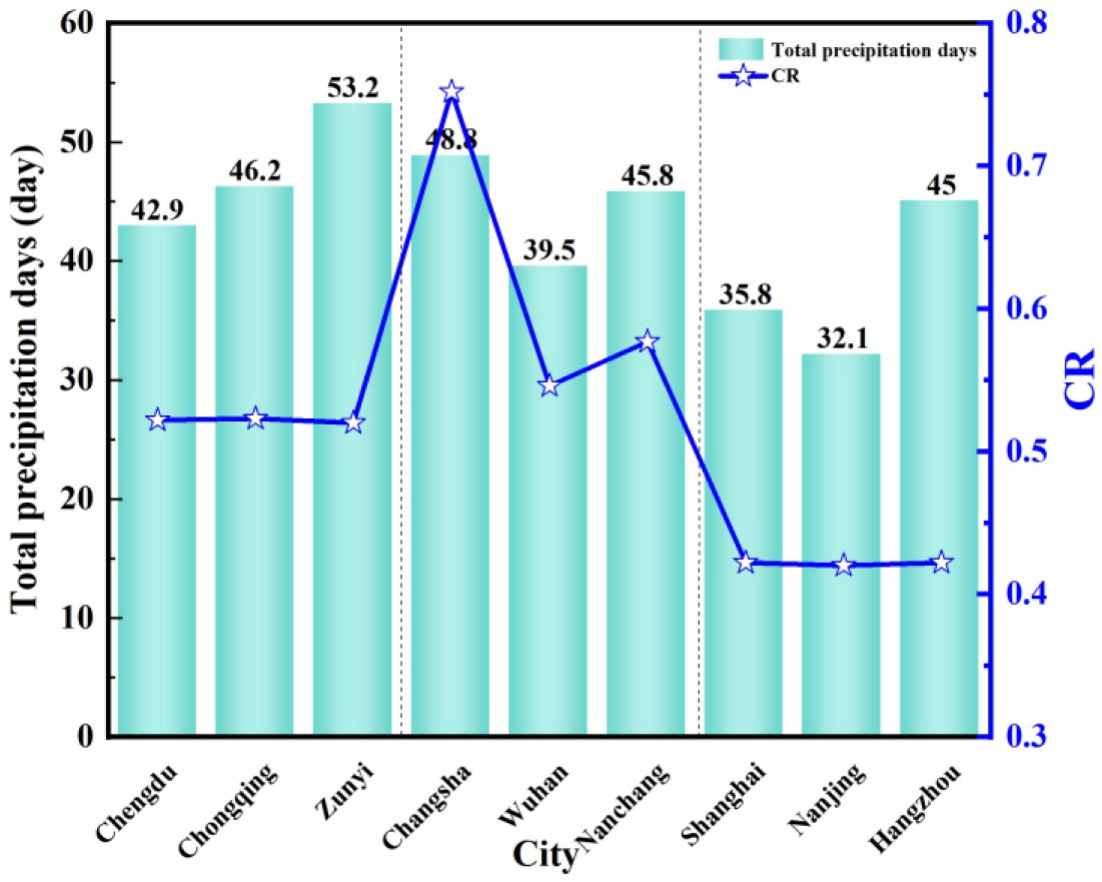
The total precipitation days and *CR* days from April to June.

Fig 18 shows the comparison chart of the total precipitation days and *CR* of nine typical cities from April to June. In the western region, the number of precipitation days is more than 40 days, of which Zunyi has reached 53.2 days. In the central region, except for Wuhan, the number of precipitation days in Changsha and Nanchang has reached more than 48 days. In the eastern region, Shanghai has reached 45 days, and the number of precipitation days in Hangzhou and Nanchang was only more than 30 days. Compared with Fig 17, although there are more precipitation days in the western region, the total precipitation is minimal, and there is no significant change in *CR*. The number of precipitation days in the eastern region has no significant relationship with *CR*. However, the *CR* increases when the number of precipitation days in the central region increases.

It shows that the total precipitation has no significant relationship with *CR*, it is affected by the geographical environment and the total number of precipitation days. The number of precipitation days in the western and eastern regions is not directly related to *CR*, and the number of precipitation days in the central region is positively correlated with *CR*. When the number of outdoor precipitation days increases, the *CR* increases. This is because there are more outdoor precipitation days in the central region, and the building is in an environment with high air humidity for a long time. The continuous water vapor leads to the continuous process of water vapor condensation into the water, and the condensation phenomenon is obvious. We can take anti-condensation measures to reduce *CR*.

### 5.5 Atmospheric pressure

**Fig 19:**
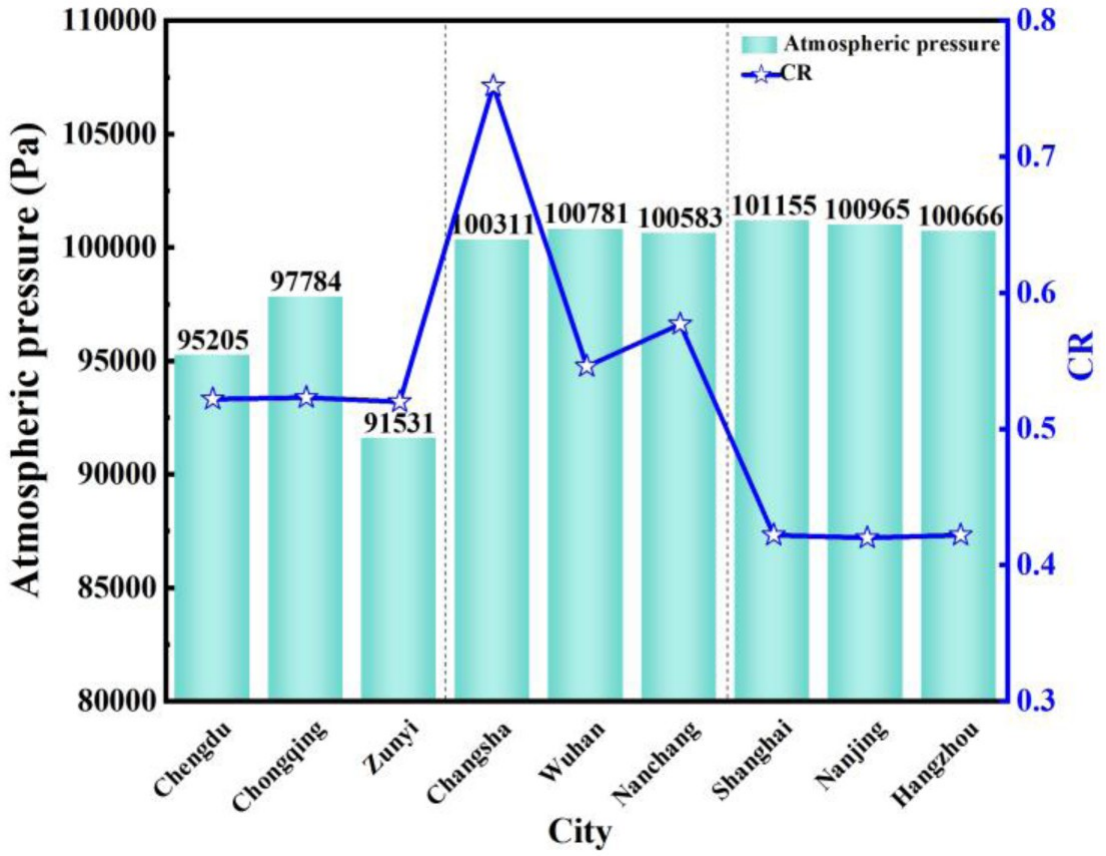
Atmospheric pressure and *CR* from April to June.

Fig 19 compares atmospheric pressure and *CR* in nine typical cities from April to June. The atmospheric pressure in the central and eastern regions is higher than that in the western region. The atmospheric pressure in the central and eastern regions is above 10000 pa, and the atmospheric pressure in the western region is lower than 10000 pa due to the higher altitude in the western region. The higher the altitude, the lower the atmospheric pressure. In addition, the atmospheric pressure is related to the altitude, atmospheric temperature, atmospheric density, Etc. The atmospheric pressure decreases with the increase in temperature. Moreover, the atmospheric pressure is related to the air composition. When the air humidity increases, the atmospheric pressure becomes lower. Therefore, atmospheric pressure is inversely proportional to humidity. When the atmospheric pressure increases, the temperature and humidity decrease.

**Fig 20:**
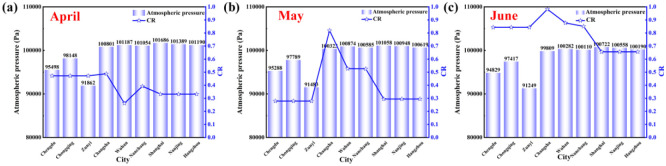
Atmospheric pressure and *CR* in April, May, and June.

Fig 20 compares atmospheric pressure and *CR* in April, May, and June in nine typical cities. The changing trend of atmospheric pressure in April, May, and June is the same, the atmospheric pressure in the central and eastern regions is higher than that in the western region, and there is no direct relationship between atmospheric pressure and *CR*. This is because the atmospheric pressure is the sum of the partial pressure of water vapor and the partial pressure of dry air, where the partial pressure of water vapor reflects the amount of water vapor content. Like altitude, it only can be used to judge the air dry and wet conditions. The atmospheric pressure affects the indoor humidity environment to a certain extent, but it is not a factor that affects condensation. It is also affected by other conditions.

### 5.6 Wind speed

**Fig 21:**
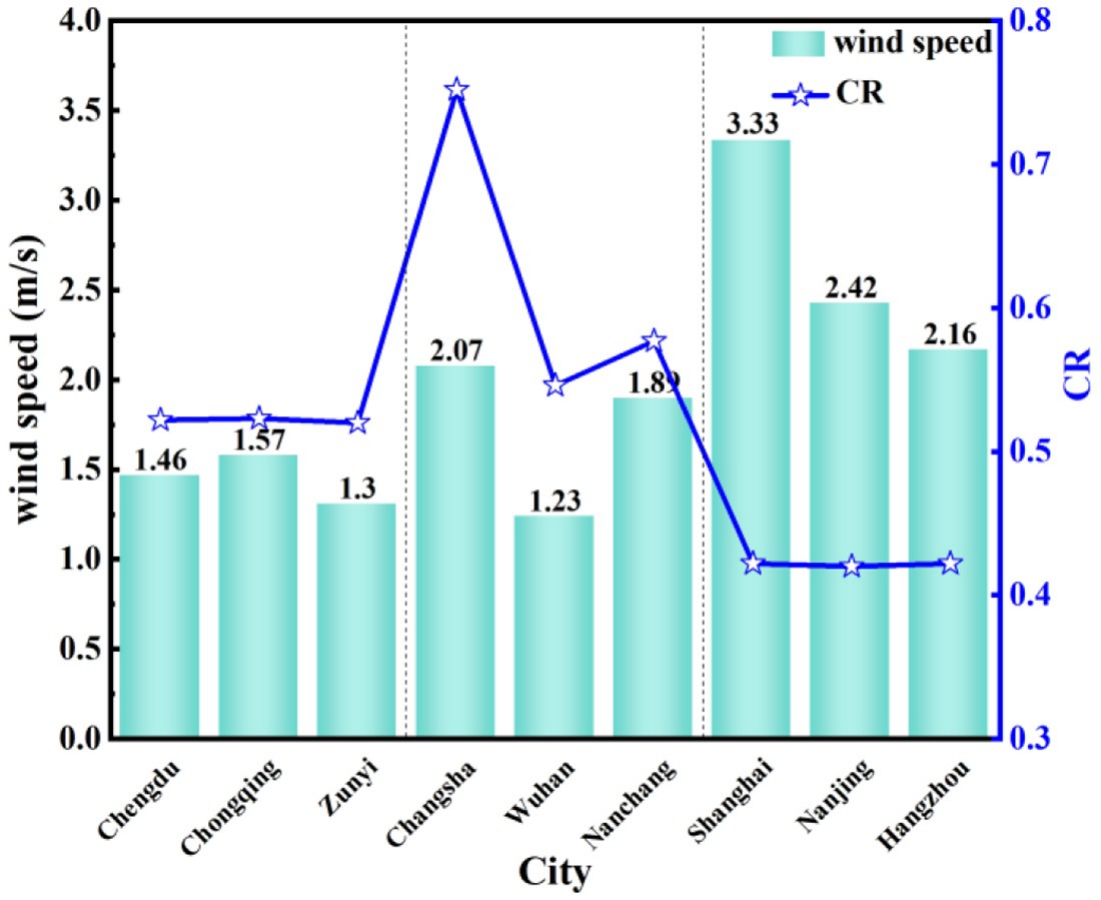
Average wind speed and *CR* from April to June.

Fig 21 shows the comparison of the average wind speed and *CR* in nine typical cities from April to June. In the western region, the wind speed is below 2 m/s, and the wind speed in Zunyi is only 1.3 m/s. In the central region, only Changsha is above 2 m/s, while the wind speed in the eastern region is higher than 2 m/s, and Shanghai even reaches 3.33 m/s. In the western and central regions, the *CR* increases when the outdoor wind speed increases. It is mainly because that cities in the eastern region are located along the coast, the outdoor wind speed is higher. When the ventilation method is natural, the outdoor air enters the room at an accelerated rate, accelerating the temperature balance. At this time, the room temperature is close to the outdoor temperature, the start time for condensation is prolonged, the duration of condensation is shortened, and the *CF*_*n*_ is reduced. When the outdoor wind speed is higher than 2.16m/s, opening the window can alleviate the condensation phenomenon on the indoor walls.

It shows that the average wind speed affects the indoor humidity environment to a certain extent. The outdoor wind speed was positively correlated with CR in the western and central regions and negatively correlated with CR in the eastern region. This is because the wind speed mainly affects the water film and air heat and mass transfer coefficient. The higher the wind speed, the greater the water film and air heat and mass transfer coefficient, and the faster the evaporation speed of the water film, thus affecting the *CR*.

## 6 Conclusion

Based on simulation of rural buildings in nine typical cities in the hot summer and cold winter region, the regularity of condensation frequency *CF*_*n*_ on the inner surface of the wall is obtained. The *CF*_*n*_ in the western, central, and eastern regions reached the highest in April, May, and June respectively. Among all the cities, Changsha has the most serious condensation on the inner surface of the wall. Analyzed from the aspects of the geographical environment (altitude) and outdoor meteorological parameters (average temperature, total precipitation and total precipitation days, relative humidity, atmospheric pressure, average wind speed). The analysis results are as follows:

From the perspective of geographical location:

(1) The altitude has little effect on the indoor humidity environment and has no direct relationship with *CR*. Other geographical factors also influence it.

From the perspective of outdoor meteorological parameters:

(2) The average temperature affects the indoor humidity environment, but it is not the main influencing factor. The average temperature in the western and eastern regions have no significant correlation with *CR*, and the outdoor temperature in the central region was positively correlated with *CR*;
(3) The relative humidity affects the indoor humidity environment and moisture condensation on the inner surface of walls, which has a strong positive correlation with *CR*;
(4) The total precipitation has no significant relationship with *CR* and is affected by the geographical environment and the total precipitation days. The total precipitation days in the western and eastern regions are not directly related to *CR*, and the total precipitation days in the central region are positively correlated with *CR*;
(5) The atmospheric pressure affects the indoor humidity environment but has no direct relationship with *CR*;
(6) The wind speed affects the indoor humidity environment to a certain extent. The wind speed was positively correlated with *CR* in the western and central regions and negatively correlated with *CR* in the eastern region.

## Supporting information

S1 Data (XLSX)

S2 Data (CSV)

S3 Data (CSV)

S4 Data (CSV)

S5 Data (CSV)

S6 Data (CSV)

S7 Data (CSV)

S8 Data (CSV)

S9 Data (CSV)

S10 Data (CSV)

S11 Data (XLSX)

S12 Data (XLSX)

S13 Data (XLSX)

S14 Data (XLSX)

S15 Data (XLSX)

S16 Data (XLSX)

S17 Data (XLSX)

S18 Data (XLSX)

S19 Data (XLSX)

S20 Data (XLSX)

S21 Data (XLSX)

S22 Data (XLSX)

S23 Data (XLSX)

S24 Data (XLSX)

S25 Data (XLSX)

S26 Data (XLSX)

S27 Data (XLSX)

S28 Data (XLSX)

S1 File (MD)

S2 File (DOCX)
