# Supplementary material for: Influence of geographical location and outdoor meteorological parameters on indoor humidity environment in rural residential buildings during the Plum Rains Season in the hot summer and cold winter region
Source: PLoS One. 2023 Oct 23;18(10):e0293181. doi: 10.1371/journal.pone.0293181 (PMC10593243; doi:10.1371/journal.pone.0293181)
Supplement: S2 File — (DOCX) [file pone.0293181.s030.docx]

# Title of Dataset: Manuscript data

The data set mainly includes: 1. Experimental verification data; 2. Simulation results data; 3. Outdoor meteorological parameters data.

## Description of the Data and file structure

The structure of the data mainly includes experimental verification, simulation results and outdoor meteorological parameters. The experimental verification data in the paper comes from the "experimental verification" in the data set, and the research on outdoor meteorological parameters in the manuscript is also included, and the corresponding data of the figures in the manuscript are given.

CR-Condensation Risk

CFn-Condensation Frequency

## Sharing/access Information

no
